# Endoscopic management of a giant ampullary cyst causing acute pancreatitis

**DOI:** 10.1055/a-2740-3403

**Published:** 2026-01-13

**Authors:** Arvind Bussetty, Michael Ma, Arvind Julius Trindade, Ankoor Patel, Petros Benias

**Affiliations:** 1 12287 Division of Internal Medicine, Rutgers Robert Wood Johnson Medical School, New Brunswick, New Jersey, United States; 2 5970 Division of Gastroenterology, Rutgers Robert Wood Johnson Medical School, New Brunswick, New Jersey, United States

Ampullary cysts (ACs) are congenital abnormalities of the gastrointestinal tract and are uncommon forms of duodenal duplication cysts 1 . They are anechoic on endoscopic ultrasound (EUS) arising from any of the three to five enteric layers with a wall of intestinal origin 2 . ACs can present with many symptoms such as duodenal/biliary obstruction, abdominal pain, infection, and pancreatitis.

We present a 17-year-old female with right upper quadrant abdominal pain, nausea, and poor oral intake found to have acute pancreatitis ( Video 1 ). On MRI, she was found to have a large cyst. Upper endoscopy with endoscopic ultrasound was notable for visualization of a large ampullary duplication cyst, not directly involving in the biliary duct ( Fig. 1 and Fig. 2 ). An attempt at cannulating the bile duct was performed which identified a false tract within the AC, likely leading to obstruction and the resultant pancreatitis. The medial aspect was cut with sphincterotome and the remainder of the AC was unroofed with snare resection ( Fig. 3 ). The ampulla was identified at the apex after resection. The bile duct and pancreatic duct were stented ( Fig. 3 ). Endoclips were placed around the edge of the dissected AC for bleeding prophylaxis. Follow-up endoscopy showed well-healed resection edges ( Fig. 4 ). The patient tolerated both procedures well. She was followed up as an outpatient and did not experience any further episodes of recurrent pancreatitis.

Endoscopic intervention of a giant ampullary cyst involving mucosectomy and stenting of the pancreatic and biliary ducts to resolve pancreatitis from biliary obstruction.Video 1

**Fig. 1:**
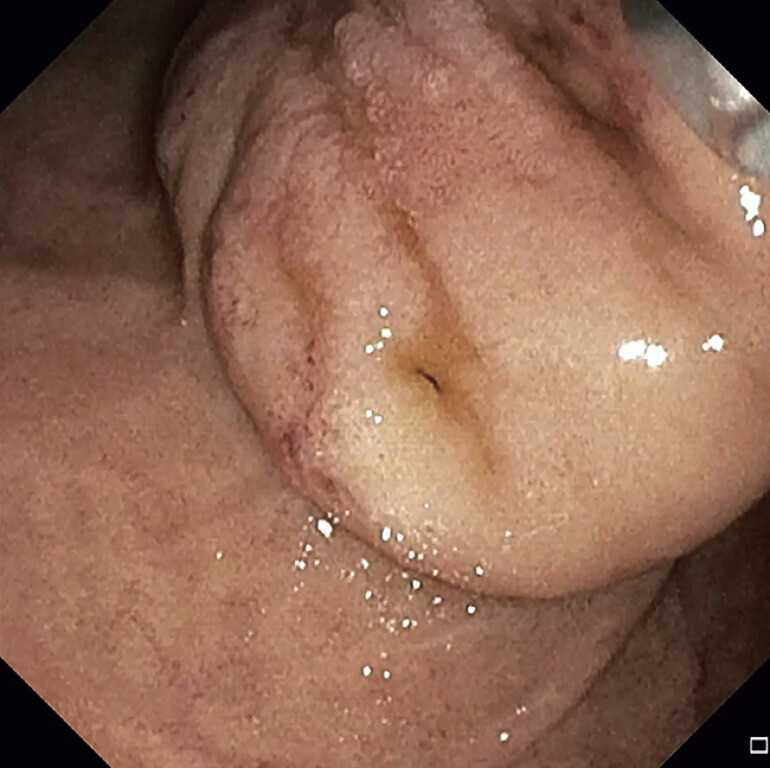
Giant cyst located at the ampulla during endoscopic intervention.

**Fig. 2:**
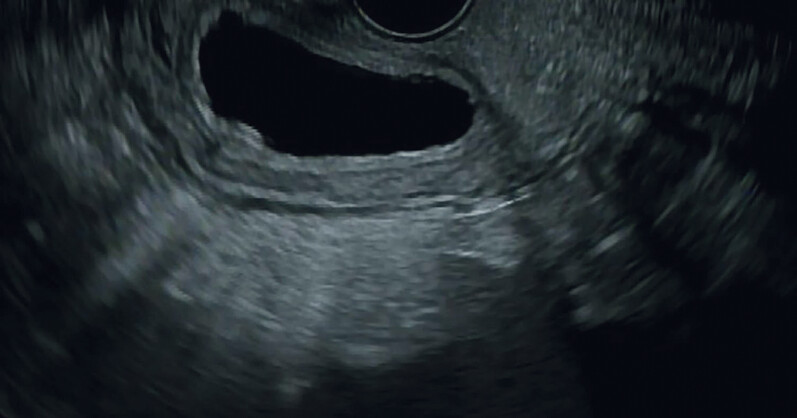
Endoscopic ultrasound demonstrating cyst with simple fluid content.

**Fig. 3:**
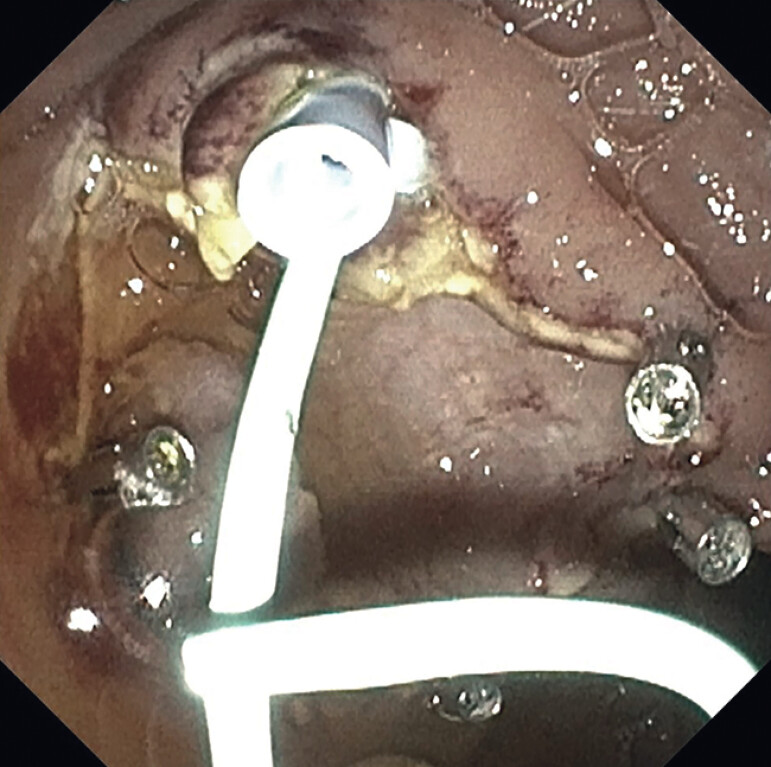
Mucosectomy of ampullary cyst completed and plastic stents placed in the biliary and pancreatic ducts.

**Fig. 4:**
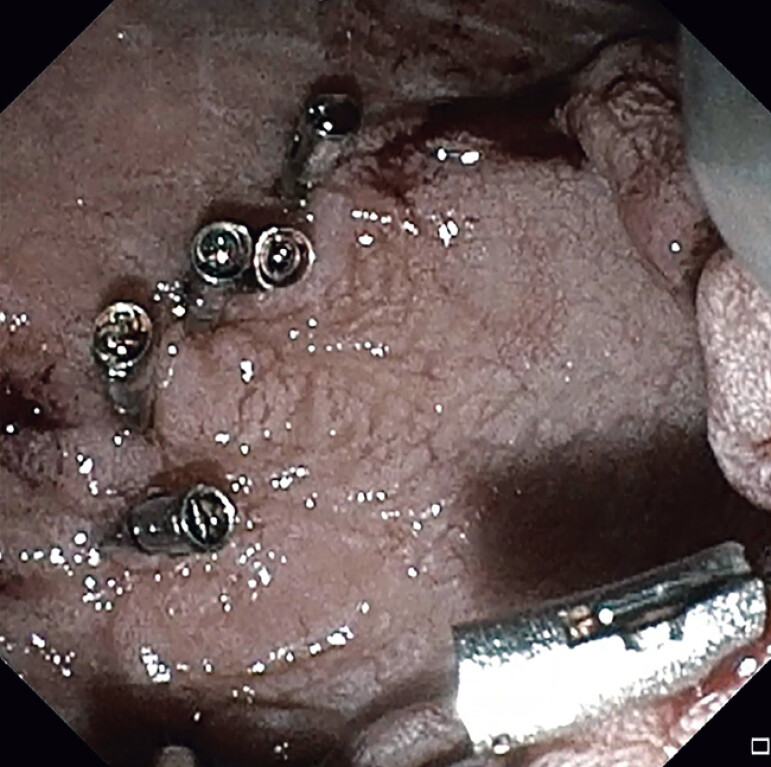
Follow-up endoscopy of mucosectomy site at ampulla, which shows well-healed edges.

Symptomatic ACs may occasionally require surgical resection 3 4 . The cyst’s location and proximity to the biliary tree rendered surgery high-risk with potential need for biliary reconstruction. This endoscopic approach avoided the morbidity of surgical intervention and resolved pancreatitis without extensive intervention.

Endoscopy_UCTN_Code_CCL_1AZ_2AK

## Notes

Endoscopy E-Videos https://eref.thieme.de/e-videos
*E-Videos* is an open access online section of the journal *Endoscopy* , reporting on interesting cases and new techniques in gastroenterological endoscopy. All papers include a high-quality video and are published with a Creative Commons CC-BY license. Endoscopy E-Videos qualify for HINARI discounts and waivers and eligibility is automatically checked during the submission process. We grant 100% waivers to articles whose corresponding authors are based in Group A countries and 50% waivers to those who are based in Group B countries as classified by Research4Life (see: https://www.research4life.org/access/eligibility/ ). This section has its own submission website at https://mc.manuscriptcentral.com/e-videos .
